# Evaluation of physical variables, thermal nociceptive threshold testing and pharmacokinetics during placement of transdermal buprenorphine matrix-type patch in healthy adult horses

**DOI:** 10.3389/fpain.2024.1373555

**Published:** 2024-03-11

**Authors:** Vaidehi V. Paranjape, Heather K. Knych, Londa J. Berghaus, Jessica Cathcart, Shyla Giancola, Hannah Craig, Caroline James, Siddharth Saksena, Rachel A. Reed

**Affiliations:** ^1^ Department of Small Animal Clinical Sciences, Virginia-Maryland College of Veterinary Medicine, Virginia Polytechnic Institute and State University, Blacksburg, VA, United States; ^2^ K. L. Maddy Equine Analytical Pharmacology Laboratory, School of Veterinary Medicine, University of California-Davis, Davis, CA, United States; ^3^ Department of Large Animal Medicine, College of Veterinary Medicine, University of Georgia, Athens, GA, United States; ^4^ Department of Civil and Environmental Engineering, Virginia Polytechnic Institute and State University, Blacksburg, VA, United States

**Keywords:** opioids, equine, analgesia, pain, pharmacology, thermal antinociception, pain-free

## Abstract

**Background:**

Matrix type transdermal buprenorphine patches have not been investigated in horses and may provide an effective means of providing continuous pain control for extended period and eliminating venous catheterization.

**Objective:**

Assessment of the physiological variables (heart rate, respiratory rate, body temperature) and thermal nociceptive threshold testing, and describing the pharmacokinetic profile of transdermal buprenorphine matrix-type patch (20 μg h^−1^ and 40 μg h^−1^ dosing) in healthy adult horses.

**Study design:**

Randomised experimental study with a Latin-square design.

**Methods:**

Six adult healthy horses received each of the three treatments with a minimum 10 day washout period. BUP0 horses did not receive a patch (control). BUP20 horses received one patch (20 μg h^−1^) applied on the ventral aspect of the tail base resulting in a dose of 0.03–0.04 μg kg^−1^ h^−1^. BUP40 horses received two patches placed alongside each other (40 μg h^−1^) on the tail base resulting in a dose of 0.07–0.09 μg kg^−1^ h^−1^. Whole blood samples (for determination of buprenorphine concentration), physiological variables and thermal threshold testing were performed before (0 h) and at 2, 4, 8, 12, 16, 24, 32, 40, 48, 56, 64, 72, and 96 h after patch application. The patches were removed 72 h following placement and were analyzed for residual buprenorphine content.

**Results:**

Between the three groups, there was no change in physiological variables across timepoints as compared to baseline (*p* > 0.1). With the higher dose, there was a significant increase in thermal thresholds from baseline values from 2 h until 48 h and these values were significantly higher than the group receiving the lower patch dose for multiple timepoints up to 40 h. 40 μg h^−1^ patch led to consistent measurable plasma concentrations starting at 2 h up to 96 h, with the mean plasma concentrations of > 0.1 ng/ml from 4 h to 40 h.

**Conclusions:**

20 μg h^−1^ and 40 μg h^−1^ patch doses were well tolerated by all horses. At higher dose, plasma buprenorphine concentrations were more consistently measurable and blunted thermal thresholds for 48 h vs. 32 h with 20 μg h^−1^ dosing as compared to control.

## 1 Introduction

In recent years, there have been significant advances in pain management for horses, with several researchers investigating pharmacology of opioid analgesics. The clinical implication of these studies is to improve the wellbeing and welfare of this species by including multimodal analgesic regimes for treatment of acute and chronic conditions. Opioids form an integral part of analgesic protocols due to their high potency and efficacy in treating different types of pain in human and veterinary medicine. Injectable µ-receptor opioid agonists such as morphine, hydromorphone and methadone are commonly used to treat perioperative pain in horses. However, clinicians hesitate to use this drug class in horses due to the apparent narrow margin between analgesia and excitation or arousal, gastrointestinal hypomotility, and challenges posed in quantifying consistent analgesic effects (1, 2).

The transdermal therapeutic system has also been assessed in horses for synthetic µ-opioid agonists such as fentanyl due to the advantage of: (i) providing noninvasive, continuous pain control for extended periods; (ii) preventing wide variations in serum drug concentrations; (iii) reducing severity of adverse effects associated with repeated post-dose peaks in plasma concentration as seen with injectable route; (iv) avoiding end of dose breakthrough pain; and (v) preventing first-pass metabolism occurring commonly with an oral route of administration (3, 4). Buprenorphine is another opioid that is available for transdermal drug delivery via patch application. This drug is a semi-synthetic, highly lipophilic oripavine derivative that is classified as a high affinity partial µ-receptor agonist and a *κ* -receptor antagonist. It's affinity for the opiate receptor is double and its potency is approximately 30 times higher than morphine. Its therapeutic response lasts much longer than other opioids and it has a wider safety profile (5–7). A transdermal matrix patch buprenorphine formulation which was initially developed for human use, has been successfully investigated in dogs (8–11), cats (12), pigs (13, 14), sheep (15, 16) and primates (17). It is evident that with transdermal buprenorphine patch, there exists a discrepancy between species with regards to nociceptive threshold testing, analgesic effects and detectable plasma concentrations. Several equine studies report the clinical utility of injectable buprenorphine (i.e., intravenous, intramuscular, subcutaneous and sub-lingual) to treat mild to moderate pain (18–24), increase nociceptive threshold (21–23), and offer superior-long lasting antinociception in comparison to butorphanol (24). To the authors' knowledge, administration of buprenorphine via transdermal patch has not been described in horses to date.

The objectives of the present study which investigated the placement of transdermal buprenorphine matrix-type patch (20 μg h^−1^ and 40 μg h^−1^ dosing) in healthy adult horses were to: (i) assess the physiological variables (heart rate, respiratory rate, body temperature) and thermal nociceptive threshold testing; and (ii) describe the pharmacokinetic profile and correlate the plasma concentrations with the level of thermal antinociception. We hypothesized that buprenorphine when delivered via the transdermal patch will: (i) minimally affect the physical examination and provide anti-nociception as detected by higher thermal threshold; and (ii) achieve quantifiable and clinically relevant plasma concentrations which will be dose dependent and correlate with the duration of thermal anticonception.

## 2 Material and methods

### 2.1 Ethics statement

This study was approved by the University of Georgia Institutional Animal Care and Use Committee (animal use protocol: A2021 03-010-Y1-A3).

### 2.2 Study animals

Six, university-owned adult, healthy horses (4 mares and 2 geldings) aged 17 ± 8 years and weighing 524 ± 44 kg were enrolled in this masked, prospective, Latin square study design. The animals were deemed healthy based on clinical history, thorough physical examination and a normal complete blood count and biochemistry profile. The entire study and all procedures took place in a temperature-controlled facility. The horses were transferred from the farm to the 3.65 m × 4.26 m stall in this facility for acclimatization 16–20 h prior to treatment administration on each occasion. During the entire duration of the study when the horses were housed in this research environment, they were provided with 0.7 kg of senior feed (Senior formula; Seminole Feed, Ocala, FL, USA) and 2–3 flakes of timothy hay twice daily with *ad libitum* access to water. On the same day, i.e., day of arrival at the facility, a 14-gauge, 13 cm intravenous catheter (DayCath; MILA International, Florence, Kentucky, USA) was placed aseptically in the cranial region of the jugular vein on the selected side for the purpose of blood collection for pharmacokinetic analysis. The horses were then weighed and a physical examination was performed to record the baseline heart rate (HR), respiratory rate (RR) and rectal temperature (Temp). The catheter was periodically flushed with heparinized saline and was monitored closely for blood clots and catheter patency.

### 2.3 Treatment groups and transdermal buprenorphine patch application

**Figure 1:**
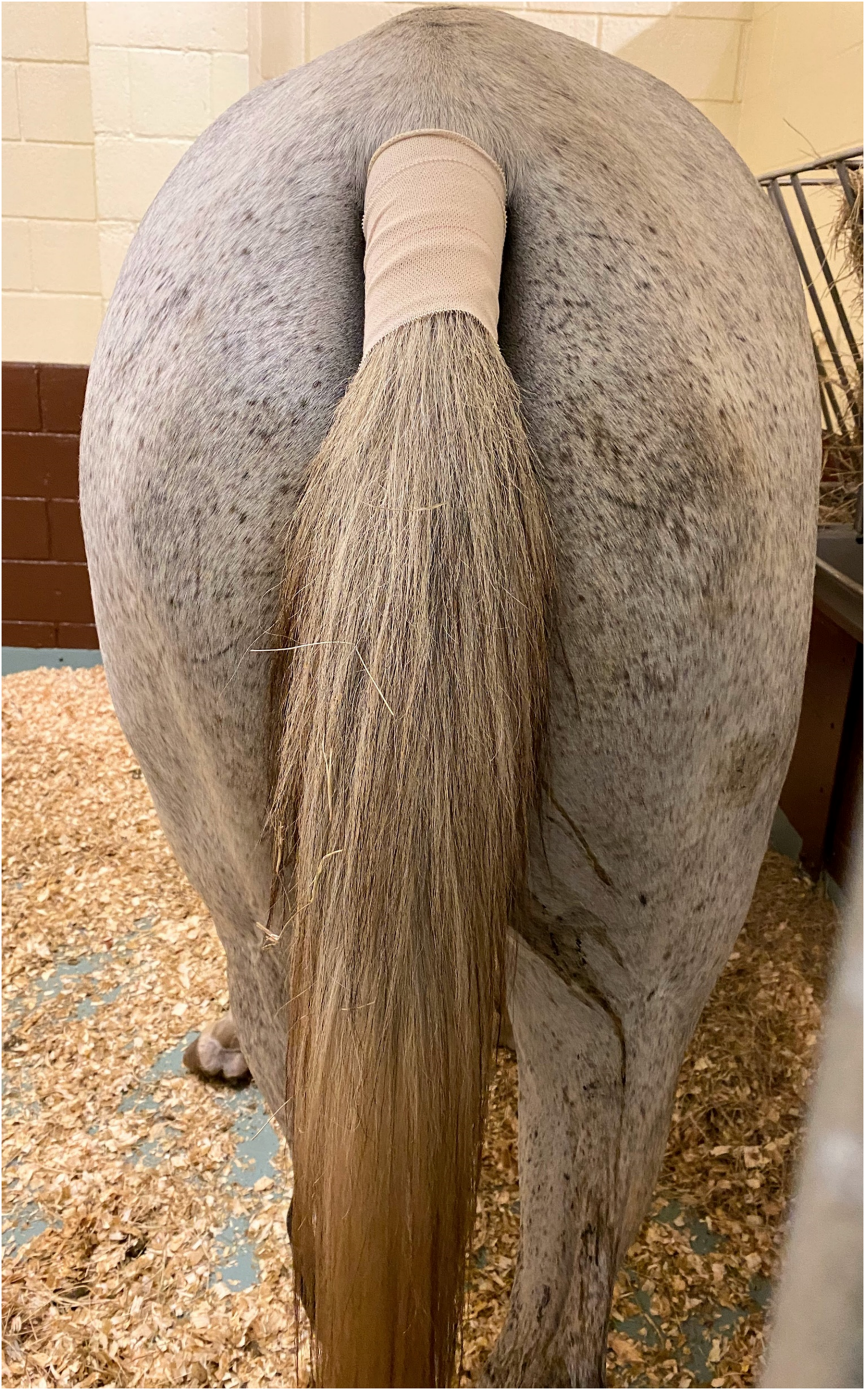
Placement of a transdermal matrix patch system containing 20 mg total buprenorphine (20 μg h^−1^; TEVA pharmaceuticals Inc., parsippany, NJ, USA) with dimensions 10.16 cm × 10.16 cm and further secured with a 3-inch porous elastic adhesive tape covering (Elastikon; Johnson & Johnson, New Brunswick, NJ, USA) on the ventral aspect of the tail base. BUP0 horses did not receive a patch, instead only the elastic adhesive tape was wrapped around the tail base. BUP20 horses received one patch (20 μg h^−1^) resulting in a dose of 0.03–0.04 μg kg^−1^ h^−1^ based on their bodyweights on the day of treatment. BUP40 horses received two patches placed alongside each other (40 μg h^−1^) resulting in a dose of 0.07–0.09 μg kg^−1^ h^−1^ based on their bodyweights on the day of treatment.

All enrolled study horses were administered all of the following three treatments and the randomization by application of Latin square was predetermined (www.randomizer.org). The washout period between treatments was a minimum of ten days. The ventral aspect of the tail base was wiped clean with a dry 10.16 cm × 10.16 cm gauze pad to remove dirt and skin debris. It was ensured that this area would allow sufficient skin to patch contact such that two patches could be situated next to each other without overlap. A transdermal patch which contained 20 mg total buprenorphine (20 μg h^−1^; TEVA Pharmaceuticals Inc., Parsippany, NJ, USA) with dimensions 7.4 cm × 7.4 cm was applied in this location and was further secured with a 7.62 cm porous elastic adhesive tape covering (Elastikon; Johnson & Johnson, New Brunswick, NJ, USA) as shown in Figure 1.

• **BUP0:** horses did not receive a patch, instead only the elastic adhesive tape was wrapped around the tail base
• **BUP20:** horses received one patch (20 μg h^−1^) resulting in a dose of 0.03–0.04 μg kg^−1^ h^−1^ based on their bodyweights on the day of treatment
• **BUP40:** horses received two patches placed alongside each other (40 μg h^−1^) resulting in a dose of 0.07–0.09 μg kg^−1^ h^−1^ based on their bodyweights on the day of treatment

### 2.4 Nociceptive thermal threshold testing

**Figure 2:**
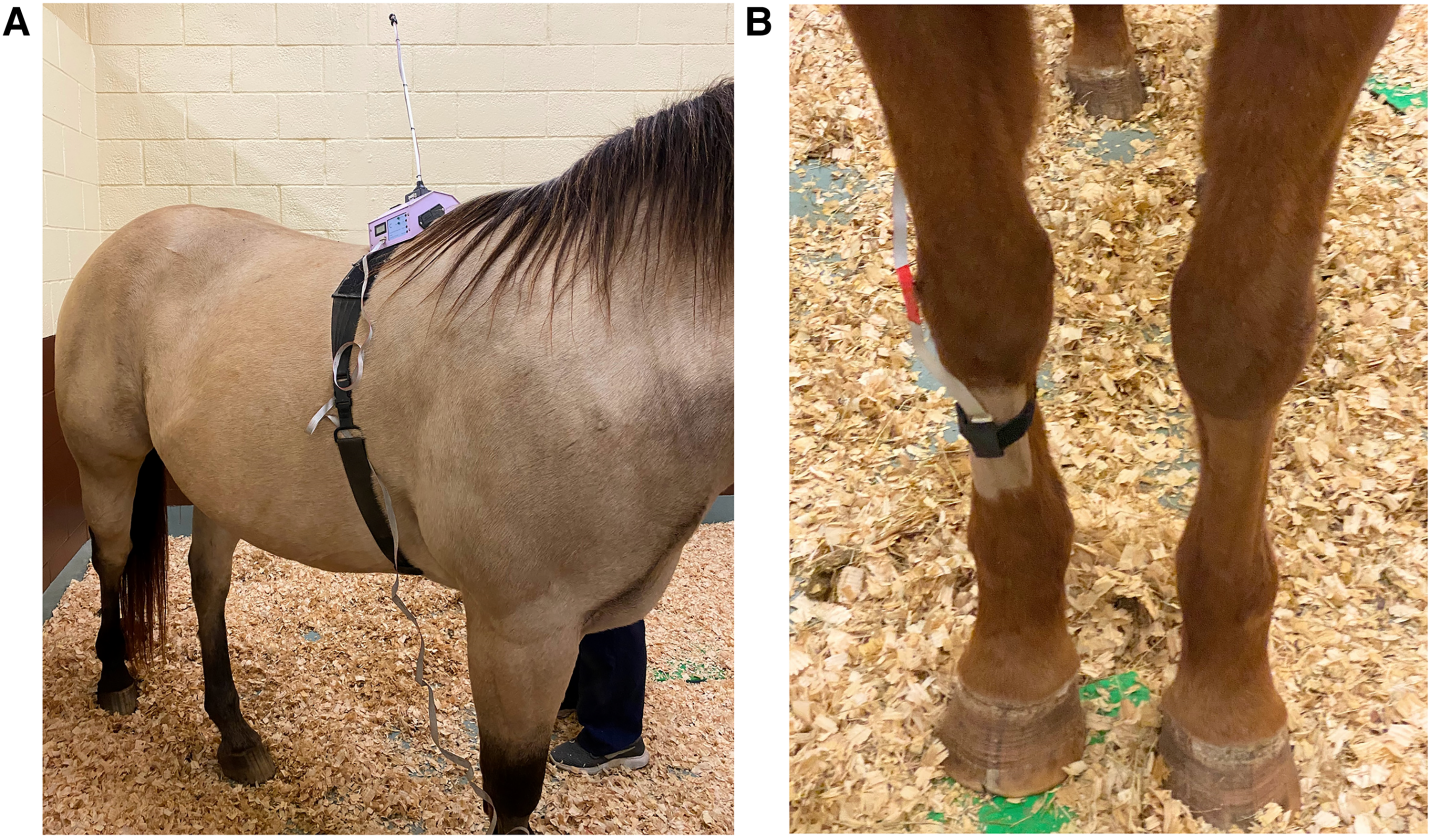
((**A**) left) Attachment of the wireless thermal threshold testing system to the horse. Cutaneous thermal threshold testing was carried out using a validated commercial wireless device (WTT2; TopCat Metrology, UK), consisting of a display control unit and heating block mounted on the horse's withers via a surcingle wrapped around the thorax with a buckle attachment. ((**B)** right) The thermal probe comprising of a heating and temperature sensing element was placed directly on the shaved area over the metacarpus and secured around the leg with the help of a nylon Velcro strap.

A coin toss aided in randomization of the metacarpus (right or left), and the skin area over the dorsal aspect was clipped with a #50 blade on the day before the treatments were administered. Cutaneous thermal threshold testing was carried out using a validated commercial wireless device (WTT2; TopCat Metrology, UK), consisting of a display control unit mounted on the horse's withers via a surcingle wrapped around the thorax with a buckle attachment (Figure 2A). The thermal element comprised of a heating and temperature sensing element was placed directly on the shaved area over the metacarpus and secured around the leg via a nylon Velcro strap (Figure 2B). Prior to each reading, the ambient temperature and skin temperature at the site of the thermal probe was documented. The masked operator standing outside the stall (VP, RR) controlled the temperature of the thermal probe via an infrared remote. The thermal element was activated by a button and heated at a rate of 0.8 °C s^−1^ until the horse reacted to the thermal stimulus by displaying avoidance behavior, i.e., pawing, stomping, lifting or rubbing their nose on the stimulated front leg (Supplementary Materials: Videos). Upon observation of an avoidance behavior following thermal stimulation, the threshold temperature for that timepoint was recorded. The unit would not heat above 55 °C and would automatically discontinue the stimulus if this temperature was reached in order to avoid thermal injury to the tissue. When the unit reached 55 °C, this was recorded as the threshold temperature for that timepoint (25–27). The Velcro strap was removed between data points and the area underneath the thermal element was examined carefully for redness and tissue damage, and the location of the thermal element on the metacarpus was varied in order to prevent tissue injury. Horses enrolled in the study had been included in previous studies utilizing thermal threshold and were accustomed to use of the device. Baseline measurements were taken in triplicate prior to patch application, with a 10-min interval between each stimulus. Thermal threshold data was then obtained at 2, 4, 8, 12, 16, 24, 32, 40, 48, 56, 64, 72, and 96 h after patch application.

### 2.5 Study timeline and data collection

**Figure 3:**
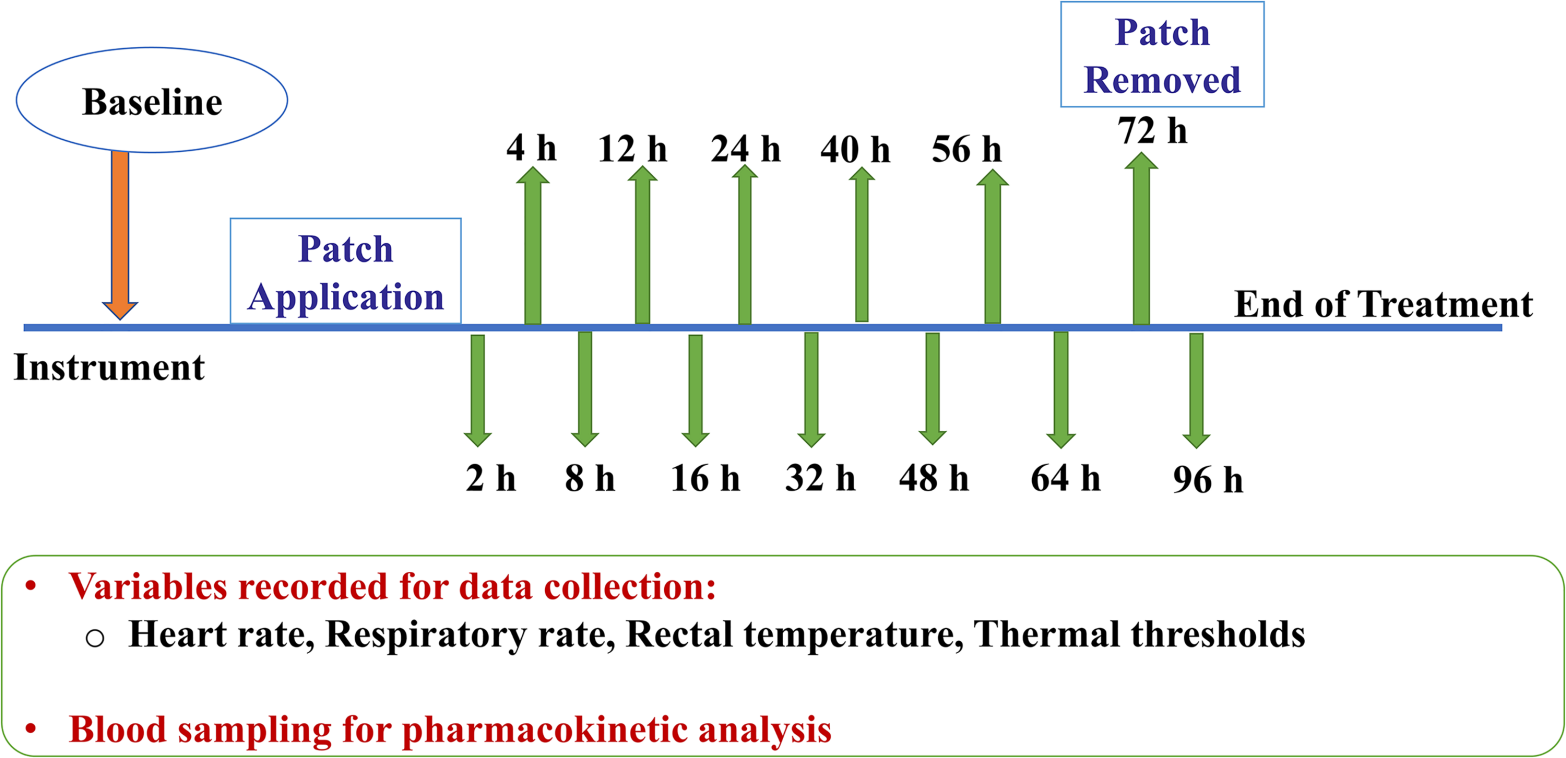
Following instrumentation, baseline data (0 h) was acquired. Dependent on the group allocated to the horse (no patch, 20 μg h^−1^, 40 μg h^−1^), the treatment was initiated. The patch and/or elastic adhesive tape was removed 72 h after treatment began. Last data collection for variables (heart rate, respiratory rate, rectal temperature and thermal thresholds) and blood sampling was performed at 96 h timepoint, which marked the end of treatment. For pharmacokinetic data acquisition, 6 ml of venous blood was drawn before (baseline or 0 h) and at 2, 4, 8, 12, 16, 24, 32, 40, 48, 56, 64, 72, and 96 h after patch application. The sampling jugular catheter and patch was removed at 72 h timepoint.

The entire timeline of the study during administration of a treatment is depicted in Figure 3. Following instrumentation, baseline data was acquired. The treatment allocated to the horse (BUP0, BUP20 or BUP40) was then applied. The patch and/or elastic adhesive tape was removed 72 h following placement. Last data collection for pharmacodynamic variables (HR, RR, rectal Temp and thermal thresholds) and blood sampling was performed at 96 h, which marked the end of data collection for that treatment. Upon completion of all three treatments, the horses were transferred back to their farm from the research facility. Once the patches were removed, they were collected in sterile bags and stored at −80 °C until later analysis of residual buprenorphine content. Whole blood was obtained for determination of buprenorphine concentration before (baseline or 0 h) and at 2, 4, 8, 12, 16, 24, 32, 40, 48, 56, 64, 72, and 96 h after patch application. A 10-ml waste sample was procured from the jugular catheter before drawing 6 ml of venous blood for buprenorphine plasma concentrations. The sampling jugular catheter was removed after 72 h, and the following blood samples were obtained by direct jugular venipuncture. Blood samples were collected in lithium heparin tubes (Green BD Hemogard™; Becton-Dickinson, Franklin Lakes, NJ, USA) and immediately underwent centrifugation at 1,300*g* for 10 min. The resultant supernatant plasma was aspirated via a 1-ml disposable pipette (Thermo Fisher Scientific, Waltham, MA, USA) and transferred to cryogenic vials (Labcon™ 1.5 ml SuperSpin™; Thermo Fisher Scientific) that were then stored at −80 °C until analysis (within 2 months of sample collection).

### 2.6 Determination of plasma concentration and pharmacokinetics

Plasma calibrators were prepared by dilution of the buprenorphine working standard solutions (Cerilliant, Round Rock, TX) with drug free equine plasma to concentrations ranging from 0.01 to 70 ng ml^−1^. Calibration curves and negative control samples were prepared fresh for each quantitative assay. Quality control samples (equine drug free plasma spiked with buprenorphine at three concentrations within the standard curve) were included with each sample.

Prior to analysis, 0.5 ml of the plasma samples were diluted with 2.0 ml 0.1 M pH 6 phosphate buffer and 0.1 ml water containing d4-buprenorphine internal standard (Cerilliant, Round Rock, TX; 40 ng ml^−1^). All samples were vortexed gently to mix, and subjected to solid phase extraction using C18UC columns 200 mg 3ml^−1^ (UCT Bristol, PA). Briefly, columns were conditioned with 2.5 ml of methanol and 3 ml of water. The samples were loaded onto the column and a minimum of 2 min was allowed for samples to pass through the column. The columns were subsequently rinsed with 2 ml 50% methanol in water, prior to eluting with 2.5 ml methanol. Samples were dried under nitrogen, dissolved in 120 µl of 10% acetonitrile (ACN) in water, with 0.2% formic acid and 40 µl injected into the liquid chromatography tandem mass spectrometry (LC-MS/MS) system.

The analyte concentrations were measured using positive heated electrospray ionization HESI(+). Quantitative analysis was performed on a TSQ Altis triple quadrupole mass spectrometer coupled with a Vanquish liquid chromatography system (Thermo Scientific, San Jose, CA). The spray voltage was 3,500 V, the vaporizer temperature was 400 °C, and the sheath and auxiliary gas were 40 and 15 respectively (arbitrary units). To optimize product masses and collision energies of each analyte, the analytes were infused into the mass spectrometer. Chromatography employed an ACE 3 C18 10 cm × 0.21 cm 3 μm column (Mac-Mod Analytical, Chadds Ford, PA) and a linear gradient of ACN in water with a constant 0.2% formic acid at a flow rate of 0.4 ml min^−1^. The initial ACN concentration was held at 10% for 0.3 min, ramped to 95% over 4.6 min and held at that concentration for 0.3 min, before re-equilibrating for 2.8 min at initial conditions.

Detection and quantification was conducted using selective reaction monitoring (SRM) of initial precursor ion for buprenorphine [mass to charge ratio (*m*/*z*) 468.3] and the internal standard d4-buprenorphine [(*m*/*z*) 472.3]. The response for the product ions for buprenorphine (*m*/*z* 101.0, 186.9, 243.0, 396.2, 414.2) and the internal standard (*m*/*z* 100.9, 186.9) were plotted, and peaks at the proper retention time integrated, using Quanbrowser software (Thermo Scientific). Quanbrowser software was used to generate calibration curves and quantitate analyte in all samples by linear regression analysis. A weighting factor of 1/X was used for all calibration curves.

#### 2.6.1 Patches analysis

For analysis, the patches were cut into 1 square cm portions and divided into two 50 ml plastic tubes. Each tube was extracted three times with 30 ml methanol by rotating for 30 min and sonicating for 5 min. The extracts were combined and brought to a final volume of 200 ml with methanol, 200 µl was then diluted to 2 ml with methanol and 100 µl of this was subjected to solid phase extraction. A volume of 20 µl was injected into the LC-MS/MS system with the analytical conditions described previously for plasma.

The response for buprenorphine was linear and gave correlation coefficients of 0.99 or better. Accuracy was reported as percent nominal concentration and precision was reported as percent relative standard deviation. Accuracy was 100% for 0.075 ng ml^−1^, 104% for 4 ng ml^−1^ and 101% for 40 ng ml^−1^. Precision was 5% for 0.075 ng ml^−1^, 3% for 4 ng ml^−1^ and 2% for 40 ng ml^−1^. The technique was optimized to provide a limit of quantitation of 0.01 ng/ml and a limit of detection of approximately 0.005 ng ml^−1^ for buprenorphine.

### 2.7 Data analysis

Numerical data such as HR, RR, rectal Temp and thermal thresholds were assessed for normality using the Shapiro-Wilk test and by observing histograms and normal Q–Q residual plots. Mixed-effects two factor analysis of variance was used to interpret the effects of time and treatment (fixed nominal effects) and the association of horse-time and horse-treatment were added as random effects. Ambient temperature, a fixed continuous effect, was also included in the model analysis for thermal threshold. To adjust for lack of sphericity, the Greenhouse–Geissner correction was applied. For making multiple comparisons with baseline measurements, the *post hoc* Tukey Honest Significant Difference test and Dunnett's test was conducted. For all analyses (SAS 9.4; SAS Institute Inc., Cary, NC, USA), *p* < 0.05 was considered statistically significant.

The peak concentration (*C*_max_) and time to peak plasma concentration (*T*_max_) were determined by visual inspection of the concentration-time data. Non-compartmental analysis and a commercially available computer software program (Phoenix Winnonlin v8.3, Certara, Princeton, NJ) were used for determination of pharmacokinetic parameters. The slope of the terminal portion of the curve, lambda *z* (*λ_z_*) was used for calculation of the half-life (HL *λ_z_*) using the equation 0.693/*λ*. The area under curve from time 0 to infinity (AUC_0 → ∞_) was determined by using the linear up log down trapezoidal rule and dividing the last measured plasma concentration by the terminal slope extrapolated to infinity.

## 3 Results

All horses successfully completed the study and patch application was well tolerated on the ventral aspect of the tail base. This site was observed to keep the patches intact in good contact with the skin and no missing data was reported due to patch dislodgement. Upon patch removal, there was no evidence of skin inflammation, papules, skin irritation or redness. All horses remained clinically healthy throughout the study and no clinically apparent adverse effects were noted at any buprenorphine dose during entire study period. Based on the subjective data during physical examination, no horses showed signs of colic with either dose or appeared excited. Overall, the horses cooperated well and stood quietly using a halter with lead rope restraint while the physical examination was being conducted.

### 3.1 Physical examination and thermal thresholds

**Figure 4:**
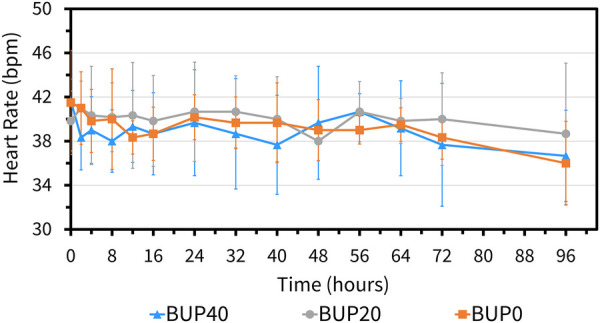
Mean ± standard deviation of heart rate values (beats per min; bpm) in six horses at various timepoints, from baseline (0 h) which coincides with before patch application to 2, 4, 8, 12, 16, 24, 32, 40, 48, 56, 64, 72, and 96 h after patch application. The three treatment groups were BUP0 (orange line with solid squares; horses did not receive a patch, instead only the elastic adhesive tape was wrapped around the tail base), BUP20 (grey line with solid circles; horses received one 20 μg h^−1^ patch resulting in a dose of 0.03–0.04 μg kg^−1^ h^−1^ based on their bodyweights on the day of treatment) and BUP40 (blue line with solid triangles; horses received two 20 μg h^−1^patches placed alongside each other resulting in a dose of 0.07–0.09 μg kg^−1^ h^−1^ based on their bodyweights on the day of treatment). The patch was removed at the 72 h timepoint.

**Figure 5:**
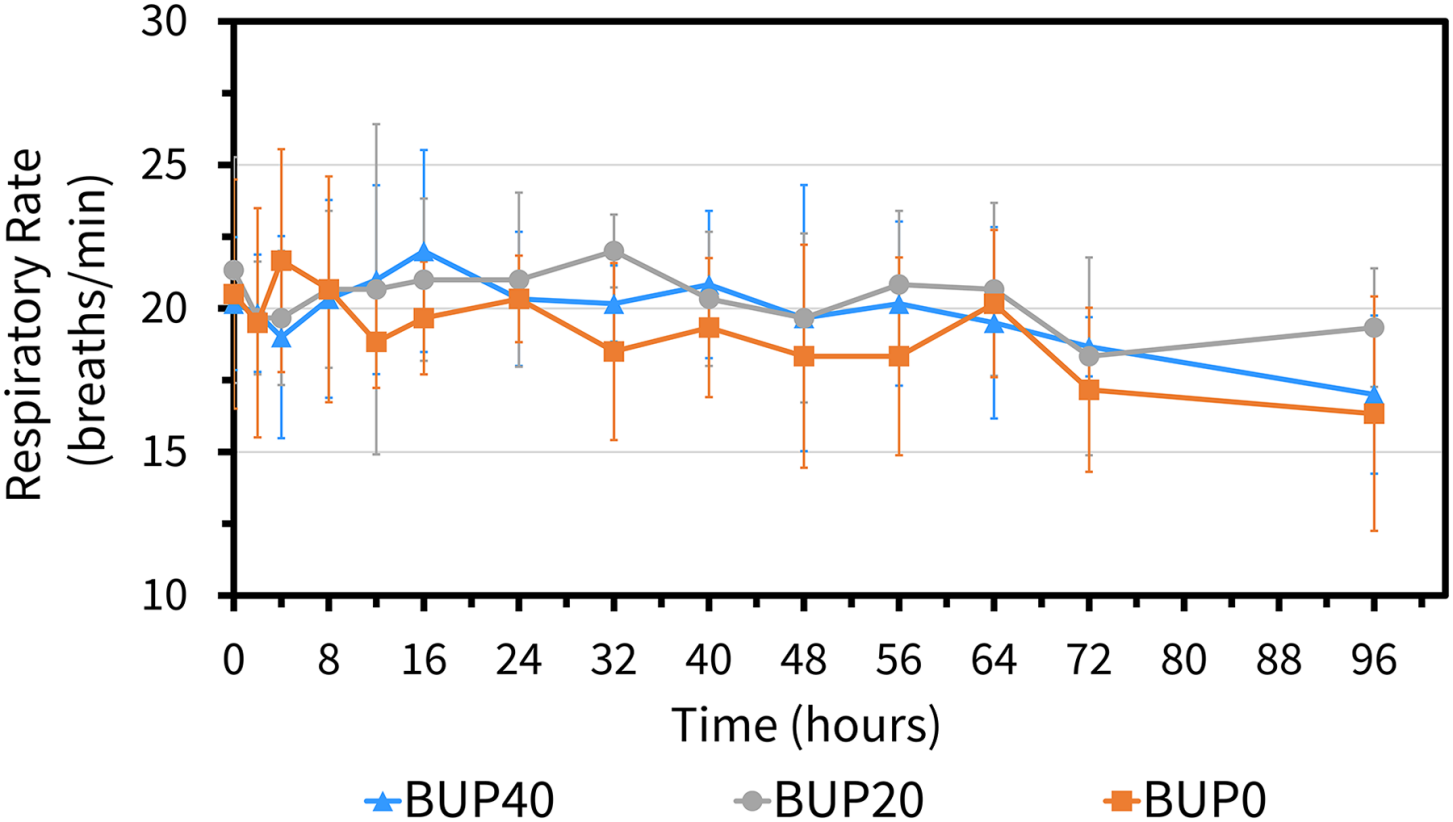
Mean ± standard deviation of respiratory rate values (breaths per min) in six horses at various timepoints, from baseline (0 h) which coincides with before patch application to 2, 4, 8, 12, 16, 24, 32, 40, 48, 56, 64, 72, and 96 h after patch application. The three treatment groups were BUP0 (orange line with solid squares; horses did not receive a patch, instead only the elastic adhesive tape was wrapped around the tail base), BUP20 (grey line with solid circles; horses received one 20 μg h^−1^ patch resulting in a dose of 0.03–0.04  μg kg^−1^ h^−1^ based on their bodyweights on the day of treatment) and BUP40 (blue line with solid triangles; horses received two 20 μg h^−1^ patches placed alongside each other resulting in a dose of 0.07–0.09 μg kg^−1^ h^−1^ based on their bodyweights on the day of treatment). The patch was removed at the 72 h timepoint.

**Figure 6:**
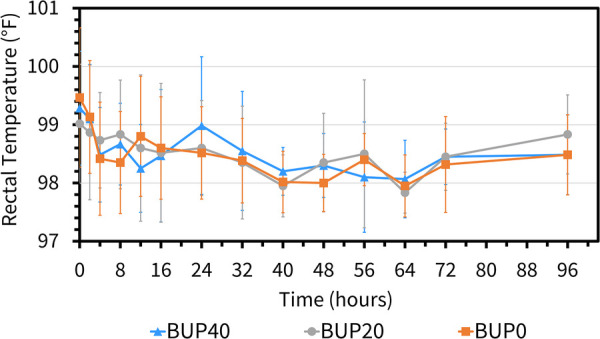
Mean ± standard deviation of rectal temperature values (degree fahrenheit; °F) in six horses at various timepoints, from baseline (0 h) which coincides with before patch application to 2, 4, 8, 12, 16, 24, 32, 40, 48, 56, 64, 72, and 96 h after patch application. The three treatment groups were BUP0 (orange line with solid squares; horses did not receive a patch, instead only the elastic adhesive tape was wrapped around the tail base), BUP20 (grey line with solid circles; horses received one 20 μg h^−1^ patch resulting in a dose of 0.03–0.04 μg kg^−1^ h^−1^ based on their bodyweights on the day of treatment) and BUP40 (blue line with solid triangles; horses received two 20 μg h^−1^ patches placed alongside each other resulting in a dose of 0.07–0.09 μg kg^−1^ h^−1^ based on their bodyweights on the day of treatment). The patch was removed at the 72 h timepoint.

The variables followed normal distribution and hence the values are represented as mean ± standard deviation. The HR at the baseline timepoint for BUP0, BUP20 and BUP40 was 41 ± 3, 40 ± 4 and 39 ± 4 beats/min, respectively. The RR at baseline timepoint for BUP0, BUP20 and BUP40 was 21 ± 4, 20 ± 3 and 20 ± 4 breaths/min, respectively. The rectal Temp at baseline timepoint for BUP0, BUP20 and BUP40 was 99.2 ± 0.97, 99.7 ± 1.2 and 98.9 ± 1.02 °F, respectively. Between the three groups, there was no change in HR (Figure 4), RR (Figure 5) and rectal Temp (Figure 6) across timepoints as compared to baseline and when compared to each other in a single horse as well as between horses (*p* > 0.1). There was no effect of treatment (*p* > 0.4) or time (*p* > 0.2), and absence of interaction between treatment and time on HR, RR and rectal Temp.

**Figure 7:**
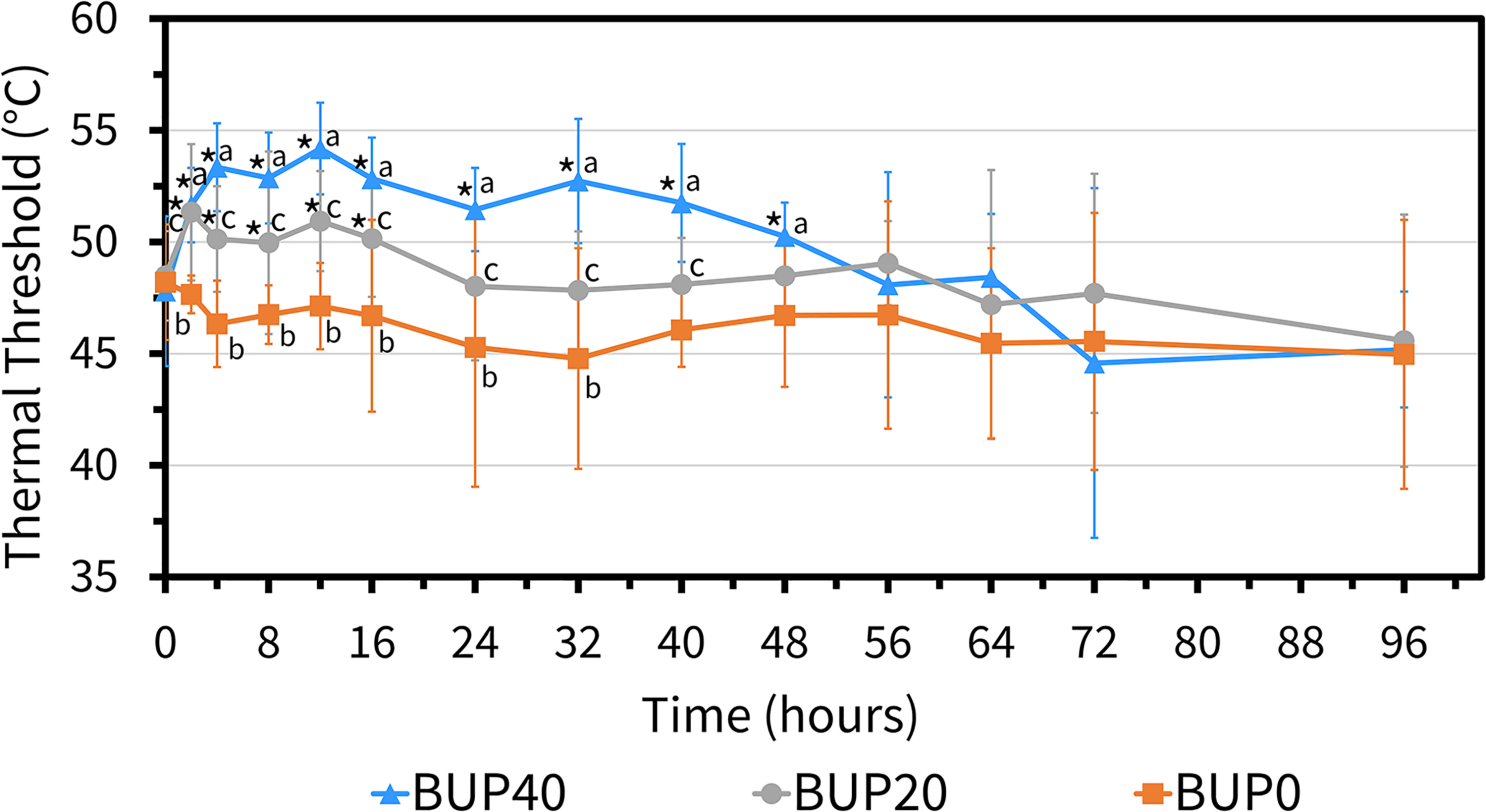
Mean ± standard deviation of thermal threshold values (degree celsius; °C) in six horses at various timepoints, from baseline (0 h) which coincides with before patch application to 2, 4, 8, 12, 16, 24, 32, 40, 48, 56, 64, 72, and 96 h after patch application. The three treatment groups were BUP0 (orange line with solid squares; horses did not receive a patch, instead only the elastic adhesive tape was wrapped around the tail base), BUP20 (grey line with solid circles; horses received one 20 μg h^−1^ patch resulting in a dose of 0.03–0.04 μg kg^−1^ h^−1^ based on their bodyweights on the day of treatment) and BUP40 (blue line with solid triangles; horses received two 20 μg h^−1^ patches placed alongside each other resulting in a dose of 0.07–0.09 μg kg^−1^ h^−1^ based on their bodyweights on the day of treatment). The patch was removed at the 72 h timepoint. *Significant difference from baseline (0 h) i.e., before patch application; ^a^Significant difference between BUP0 and BUP40; ^b^Significant difference between BUP0 and BUP20; ^c^Significant difference between BUP20 and BUP40.

During the entire experiment, ambient temperature ranged between 11.3 °C and 23.8 °C (17.3 ± 3.4 °C). The skin temperature was not different between horses undergoing each of the three treatments (*p* > 0.3) and was 29.6 ± 3.9 °C for BUP0, 29.3 ± 2.2 °C for BUP20 and 29.4 ± 2.7 °C for BUP40. There was a significant effect of treatment (*p *< 0.001) and time (*p *< 0.001), and a significant interaction between treatment and time on thermal threshold readings. With BUP40 treatment, there was a significant increase in thermal thresholds from baseline values as well as in comparison with BUP0 treatment from 2 h until 48 h (Figure 7). After this timepoint, the thermal thresholds were observed to reach the baseline values. Additionally, when horses receiving 20 μg h^−1^ and 40 μg h^−1^were compared, BUP40 thermal thresholds were significantly higher than BUP20 for multiple timepoints up to the 40 h timepoint, after which they were similar across horses. In BUP20 treatment, thermal thresholds were significantly increased as compared to BUP0 up to 32 h timepoint.

### 3.2 Pharmacokinetics

**Figure 8:**
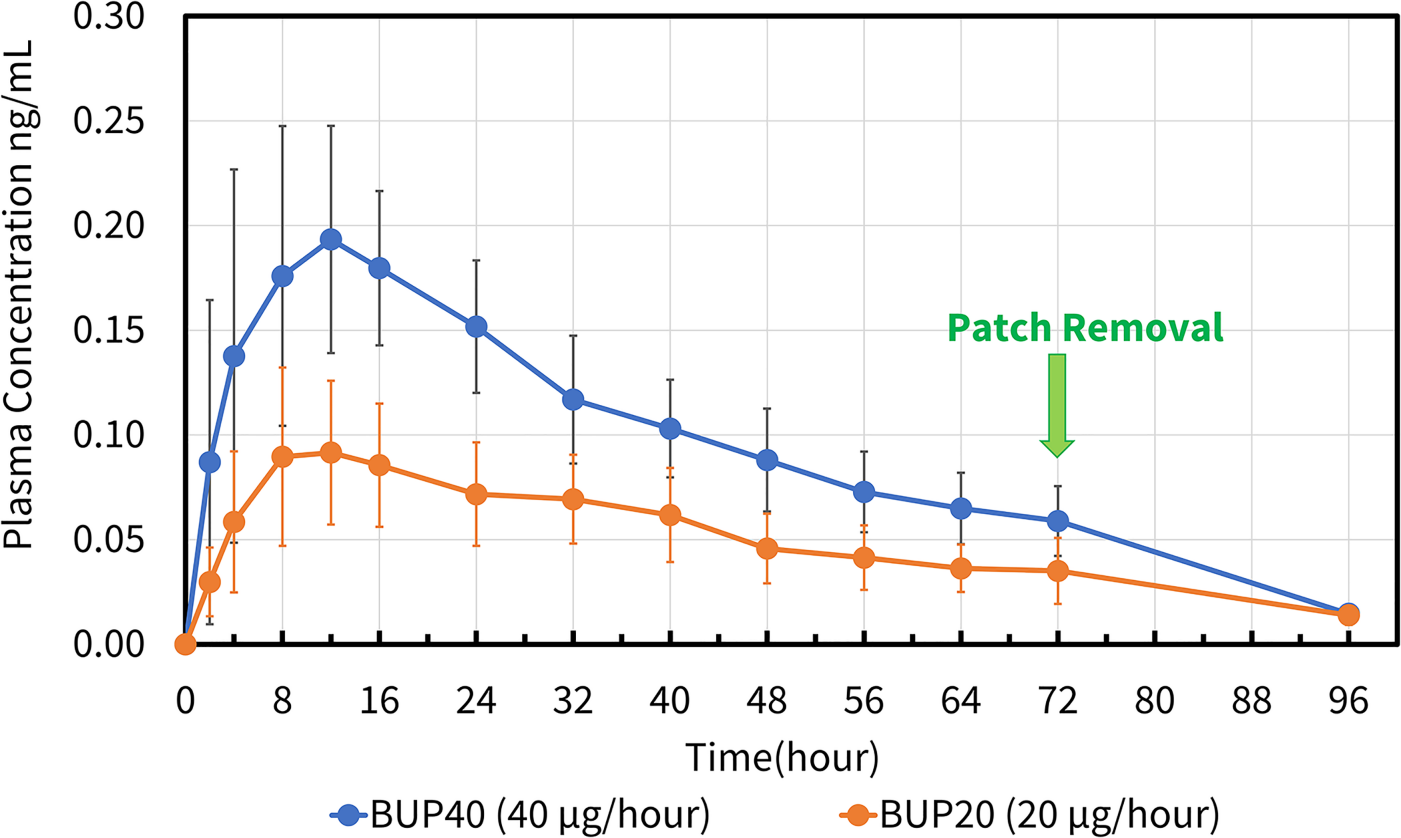
Mean ± standard deviation of plasma concentrations of buprenorphine overtime in six horses from baseline (0 h) which coincides with before patch application to 2, 4, 8, 12, 16, 24, 32, 40, 48, 56, 64, 72, and 96 h after patch application. The three treatment groups were BUP0 (horses did not receive a patch, instead only the elastic adhesive tape was wrapped around the tail base), BUP20 (orange line with solid circles; horses received one 20 μg h^−1^ patch resulting in a dose of 0.03–0.04 μg kg^−1^ h^−1^ based on their bodyweights on the day of treatment) and BUP40 (blue line with solid circles; horses received two 20 μg h^−1^ patches placed alongside each other resulting in a dose of 0.07–0.09 μg kg^−1^ h^−1^ based on their bodyweights on the day of treatment). The patch was removed at the 72 h timepoint.

Buprenorphine concentrations in horses were detected as early as 2 h and as long as 96 h in both groups (Figure 8). All six horses in BUP40 treatment showed measurable plasma concentrations starting at 2 h and persisting through the last sampling point, with the mean plasma concentrations of >0.1 ng/ml from 4 h to 40 h. The plasma concentration noted as a group mean was 0.18 ng ml^−1^ from 8 h to 16 h. In BUP20 horses, the measurable plasma concentrations were detected up to 96 h (> 0.02 ng ml^−1^ and <0.09 ng ml^−1^), and plasma concentration recorded was a group mean of 0.09 ng ml^−1^ from 8 h to 16 h timepoints. Norbuprenorphine was not detected in any horse at concentrations above the limits of detection at any time point.

The pharmacokinetic parameters generated for BUP20 and BUP40 treatments using noncompartmental analysis are presented in Table 1. The area under the curve percent extrapolation was well below 25% for both groups. When the patches were removed and submitted for analysis, the amount of buprenorphine extracted from the patch/patches was 33.5 ± 0.54 mg (83.7 ± 0.01%) in the BUP40 treatment and 17.70 ± 0.81 mg (86.5 ± 0.04%) in the BUP20 treatment.

**Table 1:** Mean ± standard deviation of pharmacokinetic parameters generated for two transdermal buprenorphine patch doses in six, adult healthy horses using noncompartmental analysis.

Pharmacokinetic Parameter	BUP20 horses receiving one patch (20 μg h^−1^)	BUP40 horses receiving two patches (40 μg h^−1^)
Tmax (h)	12.70 ± 3.93	11.33 ± 4.68
Cmax (ng ml^−1^)	0.10 ± 0.04	0.21 ± 0.06
AUC_% Extrap_Obs (%)	14 ± 14.2	3.75 ± 1.09
AUCINF_Obs (h × ng ml^−1^)	5.34 ± 1.44	9.23 ± 2.19
AUClast (h × ng ml^−1^)	4.69 ± 1.56	8.89 ± 2.15

Tmax, time of the maximum measured plasma concentration; Cmax, maximum measured plasma concentration; AUC_% Extrap_Obs, percentage of the area under the curve that has been derived after extrapolation; AUCINF_Obs, area under the curve from time of dosing to infinity; AUClast, area under the curve from the time of dosing to the last measurable concentration.

## 4 Discussion

The present study indicated that the mean plasma concentrations in horses receiving the high-dose transdermal formulations of buprenorphine (40 μg h^−1^) were >0.1 ng ml^−1^ that lasted from 4 h to 40 h of patch application and coincided with increased thermal thresholds during that entire duration. This highlights a correlation between the plasma concentrations and anti-nociceptive effect for thermal nociception in the study horses. The pattern of plasma concentrations was similar in dogs (9), cats (12), mice and rabbits (28), but significantly varied from findings in pigs (13, 14) and Cynomolgus Macaques (17). The therapeutic threshold for buprenorphine in horses has not been identified, however it has been stipulated in humans and dogs and seems to be relatively similar within species. Typically, the analgesic drug doses are based on correlation between the plasma concentration and observable anti-nociceptive effect in the face of a nociceptive stimulus that can aid in determining the therapeutic plasma concentration. By extrapolating pharmacokinetic data and evaluation of response to pain, the therapeutic plasma buprenorphine concentration threshold is 0.1–0.5 ng ml^−1^ in humans (29, 30) and 0.1–0.6 ng ml^−1^ in dogs (31, 32). While BUP40 treatment demonstrated plasma concentrations >0.1 ng ml^−1^, this was not the case in BUP20 treatment. Even though with 20 μg h^−1^ patch, measurable plasma concentrations were seen up to 96 h, the group mean was never >0.1 ng ml^−1^. Also, even though the thermal thresholds were higher than BUP0 treatment, they were significantly lower than BUP40. Once the patches were removed at the 72 h timepoint, the residual plasma concentrations dropped significantly in both groups and the thermal thresholds were similar between groups and reached baseline values. Since there were no differences found in the physical examination among BUP0, BUP20 and BUP40 treatments, both doses appeared safe and well tolerated in all horses.

Previous exploratory studies with buprenorphine in horses utilized average doses of 5–10 µg/kg via intravenous (18, 20, 21, 33–41), intramuscular (23, 24, 38, 42) and sub-lingual (40, 42, 43) routes. A common observation in most of these studies irrespective of the route used was its potential for inducing excitement, increasing spontaneous locomotory activity, decreasing gut sounds and elevating HR in healthy pain-free horses. In spite of opting for the subcutaneous route for buprenorphine administration in a few equine studies, the gastrointestinal side effects, compulsive behavior and restlessness persisted (22, 44). The doses in the present study were selected carefully on the basis of the behavioral and physiologic responses reported in these studies. We anticipated that 0.03–0.04 μg kg^−1^ h^−1^ (20 μg h^−1^) and 0.07–0.09 μg kg^−1^ h^−1^ (40 μg h^−1^) would be a safe, well tolerable dosage regime for our horses which would prevent systemic complications and excitement. Moreover, currently, the highest concentration of transdermal system available for buprenorphine in the United States is 20 μg h^−1^ and since the selected location was the ventral aspect of the tail base, placement of only two patches next to each was possible without overlap to administer 40 μg h^−1^. Future studies are imperative to evaluate whether a higher transdermal patch dose can lead to plasma concentrations lasting more than 48 h, coinciding with therapeutic drug concentrations yielding adequate analgesia, but still devoid of any systemic complications. It is also important to acknowledge that our study evaluated a one-time transdermal application of buprenorphine to combat thermal nociception, unlike some of the above-mentioned studies that investigated synergism of buprenorphine in conjugation with alpha_2_ adrenergic agonists.

During the present study, in BUP40 treatment, Cmax was 0.21 ± 0.06 ng ml^−1^, while in BUP20 treatment Cmax was 0.10 ± 0.04 ng ml^−1^. The time of the maximum measured plasma concentration was 11.33 ± 4.68 h and 12.70 ± 3.93 h in BUP40 and BUP20 horses, respectively. A single buprenorphine transdermal (hydrogel matrix technology) application in rabbits at 8.4 mg patch resulted in Cmax of 0.97 ± 0.49 ng ml^−1^ and Tmax of 3–24 h. The same type of patch in mice at 0.8 mg patch caused Cmax of 9.3 ± 1.4 ng ml^−1^ and Tmax of 1–24 h (31). Upon application of a 35 μg h^−1^ buprenorphine matrix patch in cats, plasma concentrations were 0.83 ± 0.61 ng ml^−1^ at 12 h, 1.49 ± 0.93 ng ml^−1^ at 22 h, and once the patch was removed after 72 h, they were 4.24 ± 1.31 ng ml^−1^. The Cmax was 10 ± 0.81 ng ml^−1^ and this peak occurred at times ranging from 34 h after the patch was applied to 6 h after it was removed (12). Variation in first detectable plasma buprenorphine concentrations ranging from 8 to 24 h was reported in Göttingen minipigs that underwent transdermal matrix patch application (30 μg h^−1^). Plasma buprenorphine concentrations reached a Cmax of 0.6 ± 0.1 ng ml^−1^ at a Tmax of 63.0 ± 3 h. The mean plasma buprenorphine concentration was still above 0.1 ng ml^−1^ at the last time point, i.e., 72 h (13). Another swine study did not support the use of transdermal buprenorphine patch due to large variability in drug plasma concentrations, distribution and magnitude with 35 μg h^−1^ and 70 μg h^−1^ dosing. Serum concentrations of buprenorphine were not detected in any of the animals administered the lower dose. For the high dose, Cmax was attained 12 h after application, and concentrations decreased rapidly after 18 h, while Tmax varied from 18 to 42 h with average 0.3 ± 0.2 ng ml^−1^ at 33 h (14). After a 70 μg h^−1^ patch application in dogs, buprenorphine plasma concentrations increased during the first 36 h and then remained around the 0.7–1.0 ng ml^−1^ range for the remainder of the study. A decrease in plasma buprenorphine concentration was not observed during the period of time studied. Plasma buprenorphine concentrations remained under 0.02 ng ml^−1^ in one dog (9). Evaluation of 10 μg h^−1^ and 20 μg h^−1^ transdermal buprenorphine patched in Cynomolgus Macaques indicated Cmax of 3.43 ± 1.18 ng ml^−1^ and Tmax of 57.00 ± 15.10 h for low dose and Cmax of 8.07 ± 3.85 ng ml^−1^ and Tmax of 45 ± 6 h for high dose. For 20 μg h^−1^, 0.1 ng ml^−1^ within 8 h after application for 3 of the 4 macaques was observed which remained above this plasma level for 144 h in all 4 animals. In all cases, plasma concentrations fell below the minimum by 24 h after the patch was removed (17). In pregnant sheep, maternal plasma buprenorphine concentrations expressed as median (minimum-maximum) about 26 h after a transdermal patch administration of 40 μg h^−1^ were 0.25 (0.11–1.26) μg L^−1^, while the fetal buprenorphine concentrations were 0.04 (0.01–0.07) μg L^−1^ (15).

The primary metabolite of buprenorphine is norbuprenorphine, which was undetectable following transdermal administration in the study. This analysis was in accordance with previous studies where norbuprenorphine was unmeasurable following either intravenous or sublingual route (38, 40, 42). Considering norbuprenorphine has only 25% of the intrinsic analgesic activity of buprenorphine and a low permeability into the brain, it may have minimal clinical significance (45). There is no available literature highlighting anti-nociceptive effect of norbuprenorphine in horses and hence it is uncertain whether this metabolite contributes to antinociception. It is possible that the high stability of molecular ions of norbuprenorphine may present a challenge to be detected by tandem mass spectrometry. The assay may not have the sensitivity for measuring this metabolite and this lack of optimization could affect this finding. Also, the limited metabolism of buprenorphine to norbuprenorphine may play a role. Interestingly, when the residual amount on the patches in BUP20 and BUP40 treatments was analyzed in our study, 83%–86% of the buprenorphine was still in the patch. This could be due to the patch not being in firm contact with the skin and the patch formulation altering the diffusivity of the skin lipids, sweat induced changes in skin pH or variation in skin temperature, or skin response to the pre-patch skin preparation. Skin hydration which varies in response to ambient humidity and temperature can affect integrity and barrier properties of the skin resulting in variations in the amount of drug absorbed (46, 47). The majority of buprenorphine left over in the patch explains why we saw lower plasma concentrations and hence, did not observe any significant behavioral effects, differences in the physical examination or gastrointestinal symptoms. However, it also signifies that in spite of partial drug uptake (around 14%–17%), the plasma concentrations obtained in the BUP40 treatment were >0.1 ng ml^−1^ and blunted thermal nociception for a significant period. Dependent on the literature in other species (9, 12–15, 17), once the patch is applied, there is a larger gradient between plasma and the central nervous system that is the cause of delayed increase in plasma concentrations that lead to slow transfer of buprenorphine into the central nervous system and occupying few opioid receptors. This was in contrast to our study, which saw an increase in plasma concentrations enough to counteract thermal nociception as early as 2 h and lasting up to 48 h in BUP40 horses. At <0.1 ng ml^−1^ concentration, the thermal thresholds began to return to baseline and once the patch was removed at 72 h, plasma concentrations significantly declined in the next 24 h.

In recent years, transdermal opioid delivery systems have become popular across different species and has contributed to significant advances for effective pain management via maintenance of steady blood drug concentrations over longer periods. The established transdermal opioid delivery systems are drug-in-adhesive, reservoir and matrix-type. In the present study, buprenorphine was administered via a matrix patch that includes an adhesive polymer matrix containing the drug homogenously embedded in the center. On the top of this matrix is the backing layer made up of elastomers that protects the patch from the outer environment and is impermeable to the drug. On the bottom of this matrix is the lining layer that protects the patch during storage and is peeled off before use (3, 4, 46, 47). The absence of a drug reservoir can help lower drug abuse and detrimental impact of accidental consumption. Special features that ease the crossing of buprenorphine through the skin are lower molecular weight, compact molecular structure, high lipophilicity, adequate degree of ionization, sufficient water solubility, high efficacy to restitute for limited absorption, reduced melting temperature, relatively shorter half–life, low daily dosage regime, dosing enabling absorption from a relatively small area, and matrix patches in which a total amount of a drug is localized homogenously in an adhesion layer (3). This technology ensures the release of the opioid is regulated due to the gradient concentration between the patch and the skin. Several factors can account for species-specific differences and inter-patient variability with respect to drug uptake from the patch and buprenorphine absorption via the skin such as: (i) thickness of stratum corneum and epidermis, (ii) hair density, (iii) regional blood flow, (iv) drug molecular kinetics, (v) genetics, (vi) underlying skin disease or injury, (vii) formulation of the drug-polymer matrix, (viii) skin temperature, and (ix) skin preparation (razor shaving, alcohol). The Fick's law of diffusion controls the rate of drug input from the transdermal system into the systemic circulation through skin penetration barriers, where the drug delivery is directly proportional to the drug concentration in the matrix and coefficient of drug diffusion. It is vital to note that the drug penetration into the skin is not constant and is dependent on the duration of patch application and overtime variations in cutaneous properties, available drug in the matrix and depletion of enhancers required for drug delivery (3, 48). The site chosen for patch application in the current study was the ventral aspect of the tail base, that has been previously described in horses along with the influence of patch location on uptake of fentanyl from the transdermal patch (48, 49). This is a site with limited access to the horse, thus lowering the potential for accidental removal, yet the area can easily accommodate two patches as shown in the BUP40 group. However, considering there was significant drug left behind on the patch after removal, investigating the influence of other sites such as metacarpus, gaskin, antebrachium, interscapular area is imperative to ensure the poor buprenorphine uptake from the patch is not related to the site of application.

Nociceptive threshold tests are a standard approach for assessing an anti-nociceptive effect in laboratory settings, where mechanical and/or thermal stimuli are employed to assess the efficacy of analgesics. A thermode based system was initially designed and validated in cats, however after technological advances the use of this thermal threshold testing is adapted and has gained popularity in equine studies (25–27). This well described model of thermal nociception was incorporated in our study to determine the analgesic response of different doses of transdermal buprenorphine patch on thermal thresholds. We observed a dose dependent influence on the thermal thresholds in our study horses, which is a similar occurrence previously reported with injectable opioids. This increment in the thermal thresholds was shorter lived and lasted from 2 h to 48 h timepoint in BUP40 treatment. This pattern seemed to follow trends of plasma buprenorphine concentration of >0.1 ng ml^−1^. The behavioral endpoints such as pawing, stomping, lifting or rubbing their nose on the stimulated front leg have been considered reliable for nociceptive threshold testing (25–27) and hence were carefully followed to ensure reflex related mannerisms could be differentiated from conscious perception of pain arising from the stimulated area. The horses were housed in individual stalls so that housing them close to each other wouldn't impact the results of this testing. Since there is minimal information regarding how ambient temperatures or skin temperatures can cause variation in thermal thresholds in horses, the skin temperature was not different between horses in the three treatment groups and hence doesn't seem to be a factor affecting the thermal threshold readings. The area proximal to the coronary band i.e., the mid cannon bone was chosen since it is less affected by variations in ambient and skin temperatures and blood flow that allows successful detection of behavioral endpoints (27). Gender predisposition to nociceptive sensitivity cannot be ruled out in our study.

This study has some limitations. An intravenous treatment was not included in the study design, and therefore, the bioavailability of the matrix buprenorphine was not calculated. Only a small sample size consisting of healthy, pain-free adult horses was utilized. The physiologic and behavioral effects of opioid administration can differ significantly in painful vs. non-painful animals, hence future studies in clinical patients exhibiting signs of pain are warranted. The genetic involvement for transdermal drug uptake is defined in humans, however its impact cannot be rule out in our study horses. Noxious thermal stimuli have been previously used in experimental pain models to produce superficial acute short-lasting pain, which does not best reflect visceral and somatic pain processes commonly encountered in clinical patients. Behavioral analysis and gastrointestinal function were not assessed using standards published in the literature (e.g., video footage, pedometer data, gastrointestinal motility scores, fecal and urine output, visual analog scoring, ataxia grading, sedation scores). We were not able to perform objective scoring for parameters due to the longer duration of the treatments and less available staff making it impractical. Since the undesirable effects can be of lesser magnitude in painful horses, future clinical studies are required to objectively quantify these effects and determine their association with transdermal buprenorphine patch in painful vs. non-painful horses.

## 5 Conclusion

The therapeutic approach of applying transdermal opioid patch provides various advantages such as decreasing animal stress and handling, reducing frequency of dosing, providing continuous drug delivery over an extended period, lowering the peaks and troughs in analgesic effect and the potential for less risk for systemic side effects. If undesirable events occur, the patch can be removed quickly. Following extensive literature review, this appears to be the first report of transdermal buprenorphine patch in horses. In the present study, 20 μg h^−1^ and 40 μg h^−1^ patch doses were safe and well tolerated by all horses as assessed by a physical examination. With the higher dose, there was a significant increase in thermal thresholds from baseline values from 2 h until 48 h and these values were significantly higher than the group receiving the lower patch dose for multiple timepoints up to 40 h. With the 20 μg h^−1^ patch, thermal thresholds were significantly increased as compared to baseline up to 32 h timepoint. However, the 40 μg h^−1^ patch led to consistent measurable plasma concentrations starting at 2 h up to 96 h, with the mean plasma concentrations of >0.1 ng/ml from 4 h to 40 h. Further research should aim at: (i) investigating the effect of higher dosages of the transdermal buprenorphine patch on duration of analgesia and measurable plasma concentrations, (ii) replacing the patch periodically to assess whether the plasma concentrations and analgesia are better maintained, (iii) and comparing analgesic and systemic effects in painful and non-painful horses.

## Data Availability

The original contributions presented in the study are included in the article/**Supplementary Material**, further inquiries can be directed to the corresponding author.
